# Emergent Cesarean Section in a Bandl's Ring Patient: An Obstetrics and Gynecology Simulation Scenario

**DOI:** 10.7759/cureus.3800

**Published:** 2018-12-31

**Authors:** Rahul Gupta, Emmanuel M Nageeb, Imran Minhas, Nathan Dang, Samantha A Mock, Janice Rivera, Derek A Ballas

**Affiliations:** 1 Medical Education and Simulation, Northeast Ohio Medical University (NEOMED), Rootstown, USA; 2 Emergency Medicine, Northeast Ohio Medical University (NEOMED), Rootstown, USA; 3 Osteopathy, Ohio University Heritage College of Osteopathic Medicine, Athens, USA; 4 Medical Education and Simulation, Autonomous University of Guadalajara, Guadalajara, MEX; 5 Obstetrics and Gynecology, Summa Health System, Akron, USA

**Keywords:** bandl's ring, uterine ring, contraction ring

## Abstract

Bandl’s ring is a rare pathology, although its incidence is thought to be rising. Training curricula for treating this condition is almost nonexistent. Patients who present with this disease require specific management and treatment. Practicing these techniques in a medical simulation lab allows trainees to hone their skills in a safe, inconsequential environment. We present a simulated case in which a patient presents with a Bandl’s ring.

## Introduction

Bandl’s ring, also known as a pathological uterine ring, is a constriction between a woman’s thickened upper contractile uterine segment and thinned lower uterine segment (LUS) during parturition [1, 2]. The uterine muscle creates a band in which the baby’s head or shoulders remains trapped, leading to inability to deliver the infant through the uterine incision. This constriction may result in severe trauma to the infant’s head, neck, or shoulders leading to delayed developmental milestones and cerebral palsy [3]. It is unclear what causes Bandl’s ring. It is believed that prolonged labor may play a role in the development of a constriction ring [4]. Dystocia has also been implicated as both a cause and an effect [1]. The incidence of Bandl’s ring is suggested to be 0.02% or one in every 5000 live births [5]. The literature currently provides very poor estimates of the prevalence of Bandl's rings and scholars suggest the number of cases is likely underestimated [4, 6].

Due to recent guideline changes emphasizing expectant management in the latent and active phases of labor, there has been an increase in prolonged labor for expecting mothers [7]. Thus, it is not uncommon to monitor laboring patients in the latent phase for up to 24 hours without cervical change. Because of the increase in prolonged labors, there is reasonable concern that the prevalence of Bandl’s ring has also increased. As Bandl's ring is associated with an infant mortality rate greater than 50% [3], it is paramount that proper instruction is given to healthcare providers regarding treating both the mother and child. Any delay in recognition and intervention of a Bandl’s ring can lead to an increase in mortality for the infant. Proper training and early identification significantly decrease mortality. One difficulty in providing adequate training for residents in treating Bandl’s ring is a low prevalence rate. According to the Accreditation Council for Graduate Medical Education (ACGME), the minimum requirement for an OB/GYN resident is only 145 cesarean deliveries throughout the course of his or her training [8]. Thus, it is unlikely that they will have encountered or have been properly instructed in managing this pathology. Therefore simulation is necessary in a physician's training [9].

To maximize educational benefit, features of the 12 best practices of simulation-based medical education as outlined by McGaghie et al. were incorporated in this scenario [10]. Skill acquisition and maintenance were stressed to educate trainees on proper technique in handling a Bandl’s ring. Sinha et al. found that laparoscopic surgical skills decay six months after simulation training without ongoing practice [11]. However, Crofts et al. found in obstetrics simulation that skills related to managing shoulder dystocia are maintained after 12 months [12], indicating simulation training can improve skill acquisition and retention. The conflicting studies suggest that decay depends on the skill. Evidence from various studies also shows feedback improves performance in a clinical setting and decreases procedural complications [10]. In addition, multiple studies have consistently shown simulation curricula can improve resident performance while decreasing patient errors [13-14]. The simulation in this study identified gaps in learner performance and debriefing closed these gaps through rigorous feedback with genuine inquiry and discussion.

A recent review of the literature identifies a paucity of curriculum or training materials regarding the management of Bandl's ring. The purpose of this simulation is to guide clinicians in identifying and managing a patient presenting with a Bandl's ring in order to improve patient outcomes for this high-risk low-frequency presentation.

## Technical report

**Figure 1:**
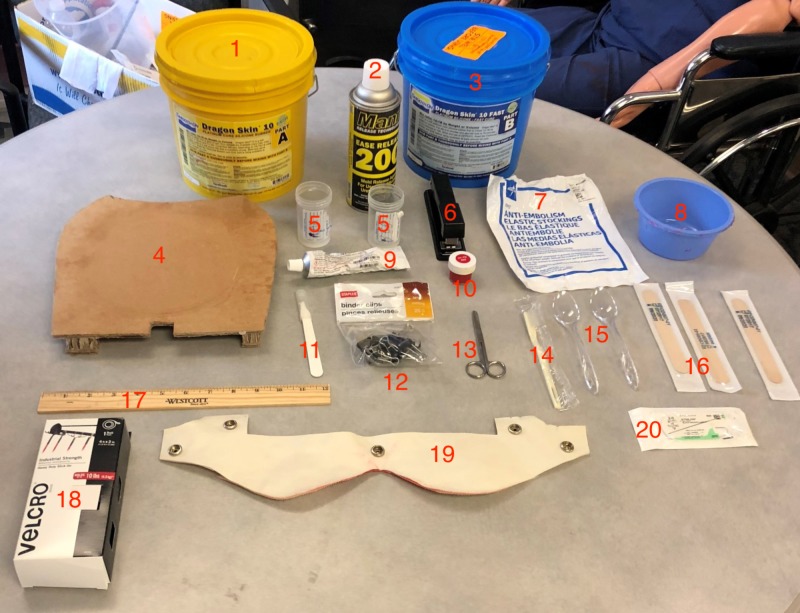
Simulated Bandl's ring materials list. From left to right: 1) Dragon Skin part A, 2) Mann Mold Release, 3) Dragon Skin part B, 4) Cardboard, 5) Vials, 6) Stapler, 7) Anti-embolism sock, 8) Mixing bowl, 9) Rubber adhesive, 10) Silc Pig Red, 11) Scalpel, 12) Binder clips, 13) Scissors, 14) Toothbrush, 15) Plastic spoons, 16) Tongue depressors, 17) Ruler, 18) Velcro, 19) Pelvic accessory, 20) Suture kit.

The Bandl’s ring scenario is run in a simulation lab made to replicate a typical obstetrics facility. The team has access to the standard tools and consultations, as well as the usual medications for a delivery. The obstetrical simulator is modified using the equipment as outlined in Figure 1. This allows for the delivering provider to make the decision to either attempt vaginal delivery or proceed to a cesarean. Fetal heart rate tracing and pertinent labs are included for review by faculty. The simulation technician provides a voiceover for verbal feedback and heightened realism. Standardization of the details of the case including history of present illness, past medical and obstetrical history, medications, and any lab work or vital signs is included. The embedded standardized nurse for the scenario works towards fulfilling any orders given as well as relaying information to the learners throughout the case.

Replication of the Bandl’s ring

Necessary Materials

1.           Mann Mond Release-Ease Release 200

2.           Medline anti-embolism elastic stockings 80% Nylon/20% Spandex

3.           Two plastic spoons

4.           Stapler/Staples

5.           50 mL Smooth-on Dragon Skin 10 Platinum Cure Silicone Rubber (Part A)

6.           50 mL Smooth-on Dragon Skin 10 Platinum Cure Silicone Rubber (Part B)

7.           Smooth-On Sil-Poxy Silicone Rubber Adhesive

8.           Smooth-On Silc Pig Red

9.           Toothbrush

10.          Piece of cardboard (23.33 cm x 27.28 cm)

11.           Mannequin’s pelvic accessory

12.          Push pins

13.          Ruler

14.          Tongue depressors

15.          Ethilon nylon suture black monofilament

16.          Two vials

17.          Mixing bowl

Application

1.           Spray the piece of cardboard with the Mann Mold Release - Ease Release 200 and let it dry for                                 five minutes (five minutes).

2.          Cut the Medline anti-embolism elastic stockings by cutting off the toe and the lower leg segment, leaving                 the foot segment only (one minute).

3.          Cut the remaining part of the sock down the middle creating a flat one level surface (one minute).

4.          Stretch and staple the stocking to the cardboard, making sure to add multiple staples per side to keep the               stocking stretched (five minutes).

5.          Use one of the plastic spoons to measure out 50 mL of Dragon Skin Part A in a vial. Care must be taken                   not to let the Part A spoon come in contact with Part B (five minutes).

6.          Repeat step five for Dragon Skin Part B (five minutes).

7.          Use a tongue depressor to ladle the Dragon Skin Part B into a mixing bowl (two minutes).

8.          Use a tongue depressor to get approximately 2 mL of Silc Pig Red and mix it with the Dragon Skin Part B                 (three minutes).

9.          Using a different tongue depressor, scoop out the Dragon Skin Part A into the mixture and mix vigorously               (three minutes).

10.        Using the tongue depressor from step nine, pour slightly under half of the combined mixture onto the                       stocking (one minute).

11.         Use the toothbrush, scrub the mixture into the stocking making sure to even the material out to be circular               with a diameter greater than 17.04 cm (four minutes).

12.        Use a tongue depressor to lift the stocking to allow the mixture to settle underneath the stocking (one                      minute).

13.        Pour the rest of the mixture on the stocking and use the toothbrush to even the mixture out. Note that the                mixture will self-level (three minutes).

14.        Use the toothbrush to pop the air bubbles in the mixture (one minute).

15.        Lift and tap the cardboard against the tabletop to bring air bubbles to the surface (one minute).

16.        Repeat step 14 (one minute).

17.        Let the mixture rest for 40 minutes to firm up (40 minutes).

18.        Use a scalpel to cut out a circular portion of the stocking with a diameter of 17.04 cm (one minute).

19.        Use a scalpel to cut a hole in the center of the disc with a diameter of 2.63 cm. The edge of the hole                         should be 7.21 cm from the edge of the disc (five minutes).

20.       Cut 10 slits around the circumference of the disc (two minutes).

21.        Use the silicone rubber adhesive to attach the slits together to form a bowl shape. Use the push pins to                    hold the glued pieces together and let sit for 15 minutes (25 minutes).

22.       Use a running stitch to attach a velcro strip to the outside of the bowl (Bandl’s ring) (two minutes).

23.       Velcro the Bandl’s ring to birthing mannequin’s pelvic accessory (Figure 2).

**Figure 2:**
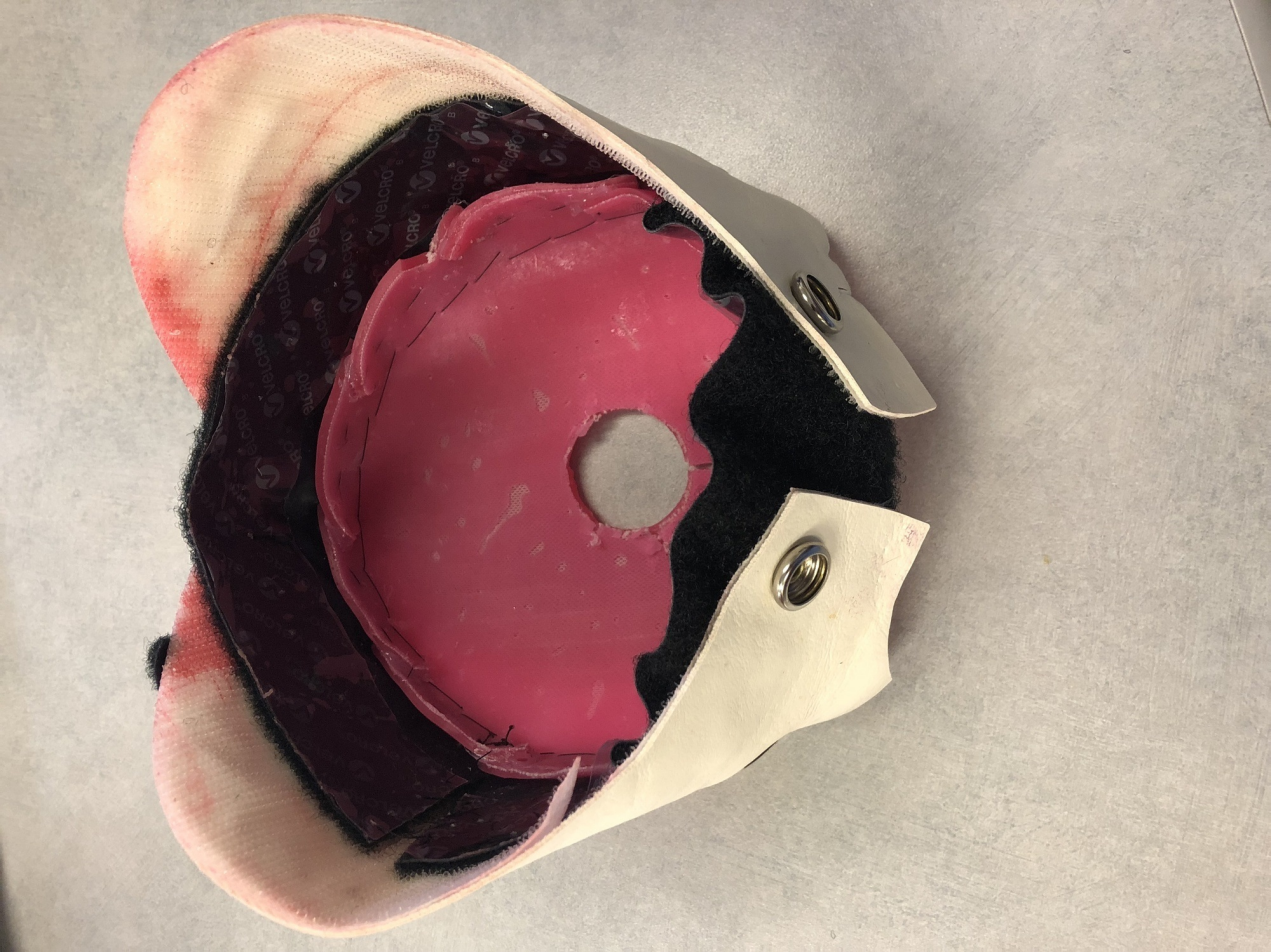
Final simulated Bandl's ring as per the materials listed in Figure 1 and replication instructions.

After the gathering of the materials is complete, creating the Bandl’s ring will take approximately two hours. The finished product will accurately portray the Bandl’s ring.

Replication of the mother’s uterus

Necessary Materials

1.      Flesh-colored duct tape

2.      Flank steak (two pack)

3.      Red biohazard bag

4.      Water

5.      Bandl’s ring

6.      Pure foam cushion by loop and threads (two pack)

7.      Mannequin

Application

**Figure 3:**
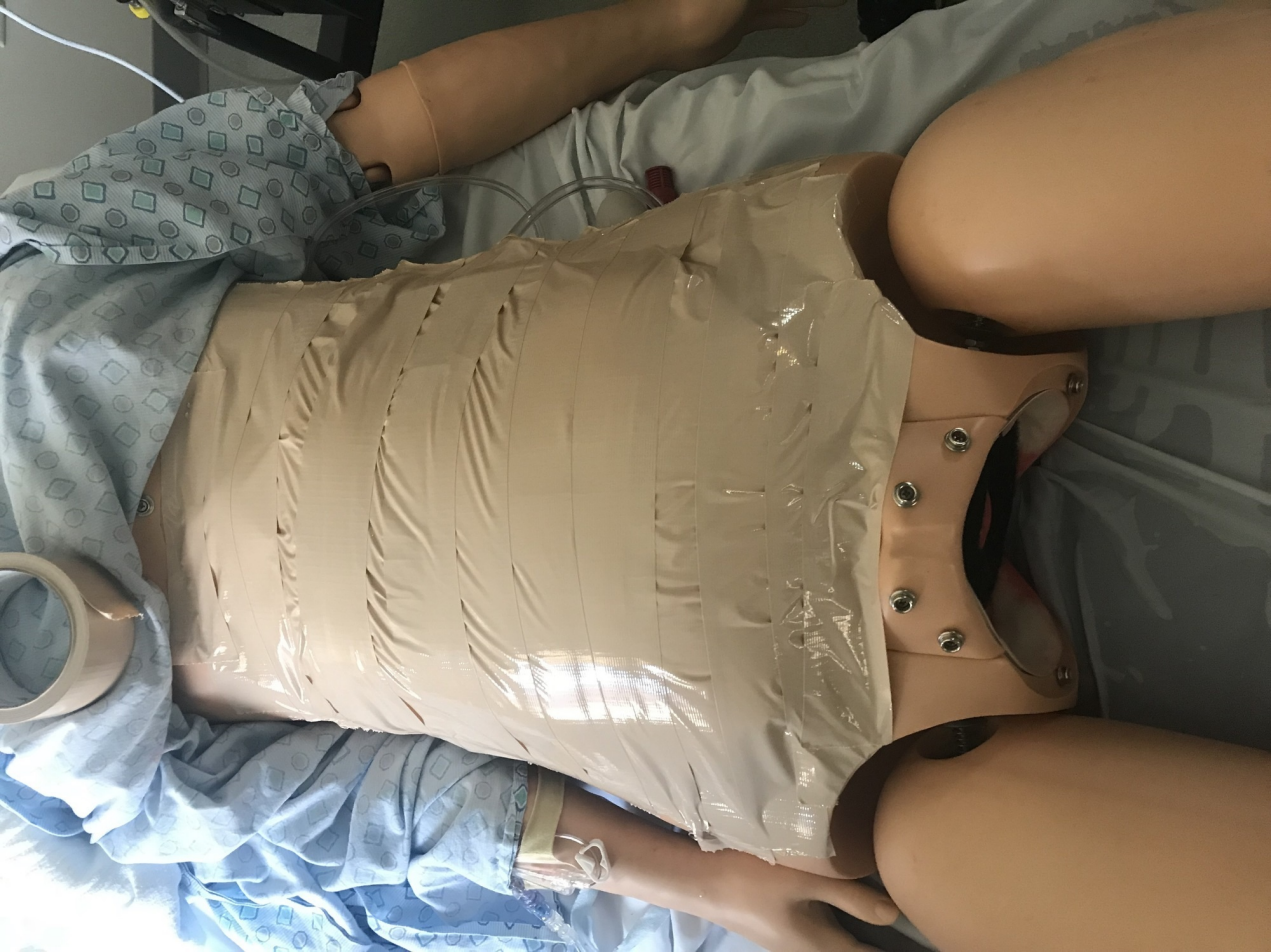
Completed mannequin with Bandl's ring, mannequin baby, and simulated uterus as per materials listed in replication instructions.

1.      Place the baby and the placenta in the biohazard bag (one minute).

2.     Place the Bandl’s ring around the bag and the baby’s head (one minute).

3.     Fill the biohazard bag with water until it barely covers the baby (one minute).

4.     Snap the Bandl’s ring into the mannequin’s pelvic area (one minute).

5.     Place the flank steak on top of the biohazard bag (one minute).

6.     Place the foam cushion on top of the flank steak and biohazard bag, cutting the foam cushion to get the best          fit (two minutes).

7.     Use the flesh-colored duct tape to cover the surface of the foam cushion (Figure 3).

After the gathering of the materials is complete, creating the pregnant woman will take approximately nine minutes. The finished product will accurately portray a pregnant woman with a Bandl’s ring complication.

Preparing the simulation scenario

**Figure 4:**
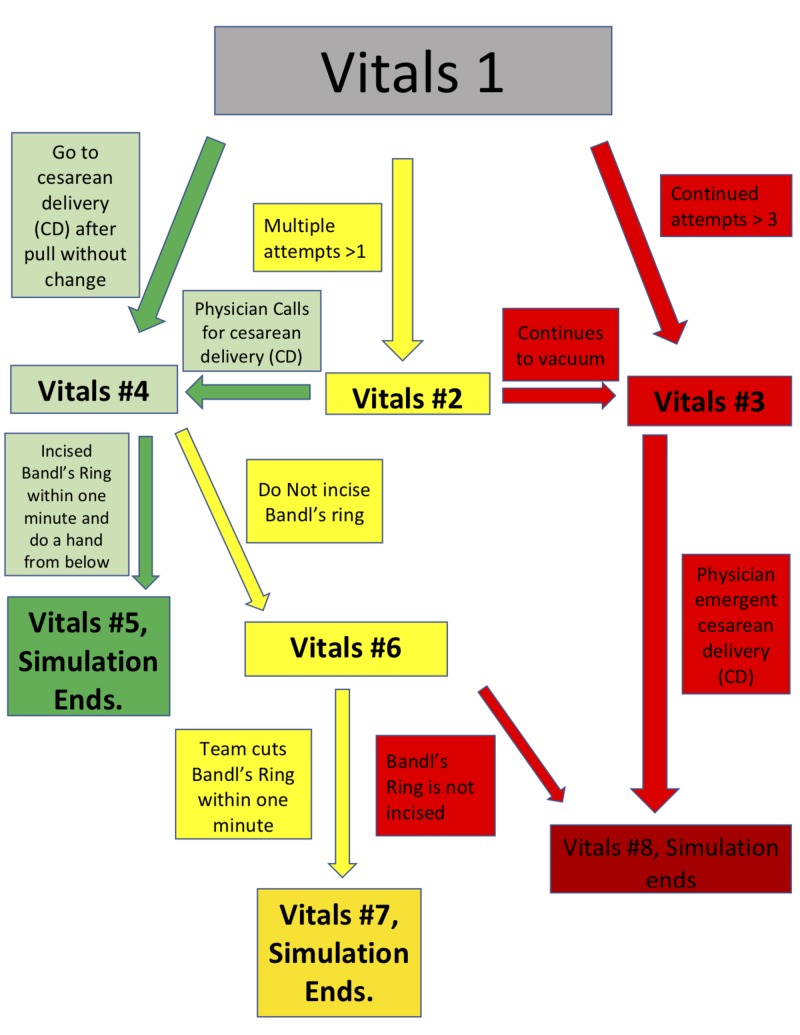
Vitals flow chart (branch points). Branch point numbers in the figure correlate with the branch points listed in Tables 1, 2.

The staff involved in the simulation includes two residents (one junior and one senior), a patient (played by our mannequin Noelle), a simulationist to provide the voice of our Noelle mannequin, a circulating nurse, the father of the baby, a certified registered nurse anesthetist, a scrub tech if available, and a simulationist doing vitals. The staff will have a team huddle before the simulation and they will be briefed on the case. They will be given the vitals and labs (Tables 1-2) as well as patient history and critical decision points. Throughout the duration of the case, the vitals will change to reflect the decisions made by the staff as outlined by the flowchart (Figure 4). Green depicts the proper decisions, red depicts the improper decisions, and yellow depicts a decision that can lead to either positive or negative results depending on the further actions of the staff.

**Table 1:** Maternal branch point vital signs. Cat: Category

	Heart Rate (Beats per Minute)	Blood Pressure (mmHg)	Temperature (^◦^F)	Respiratory Rate (Breaths per Minute)/O2 Saturation (%) (RA)	Fetal Heart Tracing
Branch Point (BP) #1	90	110/70	98.6 ^◦^F	18/98%	Cat II, baseline 140, moderate variability, episodic variable decelerations
BP #2	90	110/70	98.6 ^◦^F	18/98%	Worsening Fetal Tracing: Cat II, recurrent variables, periods of minimal variability, baseline 140
BP #3	92	105/60	98.6 ^◦^F	19/98%	Catastrophic Fetal Tracing: Cat III, baseline 60, absent variability
BP #4	92	105/60	98.6 ^◦^F	19/98%	N/A (during C-section)
BP #6	105	100/58	98.7 ^◦^F	21/95%	N/A (during C-section)

**Table 2:** Fetal branch point results.

	Appearance, Pulse, Grimace, Activity, and Respirations (APGARS)	Cord Gases
Branch Point (BP) #5	9/9	pH 7.27, Base excess 2.8
BP #7	5/7	pH 7.26, Base excess 3
BP #8	1/1/3	pH 6.9, Base Excess 14

Pre-briefing

A team huddle was held prior to the start of the simulation. In this huddle, the residents were told to treat the simulation as though it were a real-life scenario. The limitations of the mannequin were also explained as well as the roles of the staff. The resources available were shown to the students after which the students conversed and assigned roles for the upcoming simulation.

Case

A 31-year-old female presents in the delivery room with her second child (G2P1). Her last delivery was vaginal with an infant weighing eight pounds and 10 ounces at 38 weeks. Her last pregnancy delivered at term and was uncomplicated. She is currently 41 weeks gestational age and has been induced for late term. The mother has been completely dilated and pushing for three hours. The fetal head is currently at the +3 station, head position is right occiput anterior, with caput (swelling on baby head). The decision is made to attempt an operative vaginal delivery (OVD), which results/does not result in further descent.

The patient had a Bishop score of 10 at the time of induction starting 12 hours ago. She was induced with oxytocin. The fetal heart tracing is a category II for intermittent variable decelerations with pushing, but moderate variability and accelerations are present. Fetal heart rate baseline is 140 and maternal vital signs are normal. Membranes have been ruptured for six hours with clear fluid.

The patient has mild intermittent asthma and no past surgical history. She has no history of sexually transmitted diseases or abnormal pap smears. She has no relevant family history. The patient was taking prenatal vitamins and took Albuterol as needed. She has no known drug allergies.

Debrief

A team huddle was held with all members of the simulation team at the conclusion of the scenario. The discussion was shaped by points of critique and analysis of the team’s decision-making process. The staff led the discussion using advocacy-inquiry and helped the residents identify their individual strengths and weaknesses.

The crux of the deliberation centralized around identification and treatment of a Bandl’s ring complicating a cesarean delivery. The ability to safely extract the child from a Bandl’s ring without harming the child nor the mother was the focus of the debriefing.

Key branch points in the decision-making algorithm for the learners included further attempts at OVD versus proceeding with an immediate cesarean delivery. During the debrief, emphasis was placed on lack of advancement of fetal station during OVD as well as worsening fetal heart tracing. The next key point of emphasis was the time until identification of a Bandl’s ring and whether the students had an increased index of suspicion based on the patient’s labor history. At the time of cesarean, multiple delivery techniques were employed, including various hysterotomy extensions. In some of the simulations with learners, excessive force was applied in attempting to deliver the newborn. Both hysterotomy technique and appropriate use of force, including the implications of each, became crucial educational topics. Students who successfully identified the constriction ring often decided to administer tocolytic agents, but were unfamiliar with the dosages of these infrequently used medications.

Post-scenario didactics

Once the debriefing was completed, all the students took part in a 15-minute, instructor-led didactic session that reinforced the key training points during the scenario. The most important topics discussed were techniques for resolving a Bandl’s ring birth at the time of cesarean. The didactic component focused on hysterotomy technique, including a caudad-directional extension, in an attempt to release the constriction. Additional focus was directed towards differentiating this condition from an impacted fetal head. Inappropriate treatment of an impacted fetal head in this scenario can result in increased risk of skull fracture for the neonate. Risk factors were presented including prolonged labor and multifetal gestations. Medication dosages for common uterine relaxing agents to alleviate this emergency including terbutaline and nitroglycerine were reviewed. Following the lecture, the students were allotted time for questions.

## Discussion

Bandl’s ring is a relatively rare medical condition that arises in less than one percent of live births. Due to recent labor guideline changes, however, the average time expecting mothers spend in labor has increased. Therefore, it is believed that the incidences of Bandl’s rings are also rising. A pubmed search using the term “Bandl’s ring” returned only seven results. Of those seven results, none included training suggestions or curricula. This highlights the need of a standardized training simulation to prevent morbidity and mortality associated with this condition.

Since the relative prevalence of Bandl’s ring is low, it is likely that few physicians can describe the ideal management of this rare presentation. Inexperience can lead to delayed diagnosis and treatment, resulting in fetal complications such as cerebral palsy or impaired developmental milestones. Simulation thus becomes crucial for trainees to decrease unfavorable outcomes. This simulation is structured to emulate a prolonged labor, complicated by a Bandl’s ring that requires a cesarean procedure to deliver the infant without trauma. Our simulation allows for trainees to be exposed to many of the obstacles that may arise in a clinical setting and will equip them with the proper techniques to effectively treat a Bandl’s ring.

When managing this condition appropriately, the time involved to identify the Bandl’s ring is crucial. Following the identification of the condition, a cesarean delivery is required and the proper uterine relaxing agents, such as terbutaline and nitroglycerine, must be administered. The Bandl’s ring must then be timely incised, in a caudad-directional extension.

Prolonged labor and multifetal gestations are common risk factors for this condition. In an effort to communicate with patients after an emergency such as this, the value of identification of an underlying cause cannot be underappreciated. Being able to differentiate Bandl’s ring from other conditions, such as an impacted fetal head, is invaluable.

In this simulation, many of the trainees failed to recognize the presence of a Bandl’s ring in a timely manner. Those who were able to identify the ring appropriately administered tocolytic agents but failed to administer the proper dosages. Learners also consistently applied an excess of force in an attempt to deliver the baby, which could be fatal in a real-life clinical scenario.

## Conclusions

This case accentuates the significance of identifying and properly managing a Bandl’s ring in a simulation scenario. Participants are trained to effectively treat a Bandl’s ring while remaining conscious of the obstacles that may occur from inappropriate management.
